# Supplementary material for: CNNeoPP: a large language model-enhanced deep learning pipeline for personalized neoantigen prediction and liquid biopsy applications
Source: Front Immunol. 2026 Feb 4;17:1722117. doi: 10.3389/fimmu.2026.1722117 (PMC12913462; doi:10.3389/fimmu.2026.1722117)
Supplement: Supplementary file 1 [file DataSheet1.pdf]

Supporting Information

CNNeoPP: A large language model-enhanced deep learning pipeline for personalized neoantigen prediction and liquid biopsy applications

Yu Cai[1,†], Rui Chen[1,†], Mingming Song[1], Lei Wang[2], Zirong Huo[1], Dongyan Yang[1], Sitong Zhang[1], Shenghan Gao[3], Seungyong Hwang[4], Ling Bai[5], Yonggang Lv[3*], Yali Cui[1*], Xi Zhang[5*]

[1] College of Life Sciences, Northwest University, Xi'an, Shaanxi, People's Republic of China.

[2] Institute of Nuclear and New Energy Technology, Tsinghua University, Beijing, People's Republic of China.

[3] Department of Thyroid Breast Surgery, Xi'an NO.3 Hospital, the Affiliated Hospital of Northwest University, Xi'an, Shaanxi, People's Republic of China.

[4] Department of Statistics and Research Institute of Applied Statistics, Chonbuk National University, Jeonbuk, Jeonbuk-do, Republic of Korea.

[5] Department of Ophthalmology, The Second Affiliated Hospital of Xi'an Jiaotong University, Xi'an, Shaanxi, People's Republic of China.

[†] These authors have contributed equally to this work and share first authorship

[*] Corresponding authors: Yonggang Lv (lvygwytt@163.com), Yali Cui (yalicui@nwu.edu.cn), Xi Zhang (xzhangtx@gmail.com).

Derivation of immunogenicity-associated features

To define features associated with neoantigen immunogenicity, a comprehensive literature review was conducted. Eleven numerical features (F1–F11) were identified and calculated for each peptide–HLA pair as described below:

Proteasomal Cleavage, TAP Transport Efficiency and NetCTLpan Score: These three features were calculated using NetCTLpan-1.1(1), providing estimates of proteasomal cleavage likelihood, TAP transport efficiency, and a composite NetCTLpan processing score.

Peptide-HLA Binding Affinity: Binding affinities for mutant and wild-type peptides were predicted using NetMHCpan-4.1(2), with results expressed as %rank values. Peptides with %rank < 0.5 were classified as strong binders, and those with %rank < 2 were defined as weak binders.

Peptide-HLA Binding Stability: Binding stability was predicted using NetMHCstabpan-1.0, and reported as predicted stability (Pred) values(3).

Peptide Hydrophobicity: Hydrophobicity was calculated by summing the Kyte–Doolittle values of all amino acids within the mutant peptide sequence (AAindex JURD980101).

T-cell Contact Residues Hydrophobicity: Hydrophobicity scores were also calculated specifically for the T-cell contact region (residues at positions 4 to n–1, excluding anchor residues), using the same hydrophobicity scale.

Peptide Weight: The molecular weight of each peptide was determined by summing the monoisotopic masses of individual residues and subtracting the mass of water (18.015 Da) per peptide bond.

Peptide Entropy: Entropy scores were calculated by summing residue-specific values from AAindex HUTJ700103.

IEDB Immunogenicity Score: Immunogenicity scores were obtained using the IEDB Immunogenicity Prediction Tool, which evaluates amino acid composition at T-cell receptor contact positions (http:// tools.iedb.org/immunogenicity/).

Agretopicity: This metric was calculated as the ratio of binding affinity (IC50) between the mutant and corresponding wild-type peptide–HLA complexes, representing differential binding potential.

Processing of DNA-seq and RNA-seq data

Whole-exome sequencing (WES) data underwent quality control using FastQC (v0.12.1)(4). Adapter sequences and low-quality bases (Phred < 33) were removed using Trimmomatic (v0.39)(5).

Reads were aligned to the hg38 human genome using BWA (v0.7.12)(6). Resulting BAM files were sorted using SAMtools, and PCR duplicates were removed with Picard's MarkDuplicates. Base quality score recalibration (BQSR) was performed using GATK (v4.4)(7). RNA-seq data were quality-filtered using Cutadapt, with adapters removed and bases with Phred scores < 25 trimmed to ensure high integrity for expression quantification.

Somatic mutation calling and filtering

Somatic SNVs were identified using Mutect2 (GATK v4.4) in paired tumor–normal mode. BQSR-processed BAM files were used, and germline variants were filtered by applying a germline resource file. Cross-sample contamination was estimated using GetPileupSummaries and CalculateContamination, generating contamination tables for filtering. Mutations were filtered using FilterMutectCalls, and those labeled as "PASS" were retained with SelectVariants for downstream neoantigen prediction.

Mutation annotation and peptide extraction

Somatic mutations were annotated using ANNOVAR, focusing on nonsynonymous variants. A custom Python (v3.9.12) script was used to translate each variant into a 21-mer mutant peptide sequence centered on the mutation, paired with the wild-type sequence. These were then fragmented into 8–11-mer peptides, ensuring the mutation was retained within each fragment to conform to MHC class I binding requirements.

Gene expression quantification and filtering

Gene isoform expression levels were quantified using Kallisto(8), with the reference transcriptome from Ensembl GRCh38. An index was generated for quantification, and expression was reported in transcripts per million (TPM). Only peptides derived from transcripts with TPM $\geq$ 1 were retained, ensuring biological relevance in downstream prediction.

HLA genotyping and binding affinity prediction

HLA class I alleles were inferred from normal WES data using OptiType(9), which achieves ~97% genotyping accuracy(10). Binding affinities for mutant and wild-type peptides were predicted using NetMHCpan-4.1(11). Peptides with a %rank > 2 were excluded to retain only strong and weak binders likely to be presented by HLA class I molecules.

Immunogenicity prediction using CNNeo

Candidate neoantigens, their HLA types, and all 11 computed features were input into the CNNeo model for immunogenicity prediction. Each peptide–HLA pair was assigned a numeric immunogenicity score, which quantitatively reflects the likelihood of eliciting a T-cell response. Higher scores indicated a greater predicted immunogenic potential. These scores were used to rank candidate peptides, enabling systematic prioritization for downstream analysis, validation, and integration into CNNeoPP.

References

1. Stranzl T, Larsen MV, Lundegaard C, Nielsen M. Netctlpan: Pan-Specific Mhc Class I Pathway Epitope Predictions. *Immunogenetics* (2010) 62(6):357-68. Epub 2010/04/10. doi: 10.1007/s00251-010-0441-4.
2. Jurtz V, Paul S, Andreatta M, Marcatili P, Peters B, Nielsen M. Netmhcpan-4.0: Improved Peptide-Mhc Class I Interaction Predictions Integrating Eluted Ligand and Peptide Binding Affinity Data. *Journal of Immunology* (2017) 199(9):3360-8. Epub 2017/10/06. doi: 10.4049/jimmunol.1700893.
3. Rasmussen M, Fenoy E, Harndahl M, Kristensen AB, Nielsen IK, Nielsen M, et al. Pan-Specific Prediction of Peptide-Mhc Class I Complex Stability, a Correlate of T Cell Immunogenicity. *Journal of immunology (Baltimore, Md : 1950)* (2016) 197(4):1517-24. Epub 2016/07/13. doi: 10.4049/jimmunol.1600582.
4. de Sena Brandine G, Smith AD. Falco: High-Speed Fastqc Emulation for Quality Control of Sequencing Data. *F1000Res* (2019) 8:1874. Epub 2019/11/07. doi: 10.12688/f1000research.21142.2.
5. Bolger AM, Lohse M, Usadel B. Trimmomatic: A Flexible Trimmer for Illumina Sequence Data. *Bioinformatics* (2014) 30(15):2114-20. Epub 2014/04/04. doi: 10.1093/bioinformatics/btu170.
6. Li H, Durbin R. Fast and Accurate Short Read Alignment with Burrows-Wheeler Transform. *Bioinformatics* (2009) 25(14):1754-60. Epub 2009/05/20. doi: 10.1093/bioinformatics/btp324.
7. Van der Auwera GA, Carneiro MO, Hartl C, Poplin R, Del Angel G, Levy-Moonshine A, et al. From Fastq Data to High Confidence Variant Calls: The Genome Analysis Toolkit Best Practices Pipeline. *Current protocols in bioinformatics* (2013) 43(1110):11.0.1-.0.33. Epub 2014/11/29. doi: 10.1002/0471250953.bi1110s43.
8. Bray NL, Pimentel H, Melsted P, Pachter L. Near-Optimal Probabilistic Rna-Seq Quantification. *Nat Biotechnol* (2016) 34(5):525-7. Epub 2016/04/05. doi: 10.1038/nbt.3519.
9. Szolek A, Schubert B, Mohr C, Sturm M, Feldhahn M, Kohlbacher O. Optitype: Precision Hla Typing from Next-Generation Sequencing Data. *Bioinformatics* (2014) 30(23):3310-6. Epub 2014/08/22. doi: 10.1093/bioinformatics/btu548.
10. Cai Y, Chen R, Gao S, Li W, Liu Y, Su G, et al. Artificial Intelligence Applied in Neoantigen Identification Facilitates Personalized Cancer Immunotherapy. *Frontiers in Oncology* (2022) 12:1054231. Epub 2023/01/27. doi: 10.3389/fonc.2022.1054231.
11. Jurtz V, Paul S, Andreatta M, Marcatili P, Peters B, Nielsen M. Netmhcpan-4.0: Improved Peptide-Mhc Class I Interaction Predictions Integrating Eluted Ligand and Peptide Binding Affinity Data. *Journal of immunology (Baltimore, Md : 1950)* (2017) 199(9):3360-8. Epub 2017/10/06. doi: 10.4049/jimmunol.1700893.
