# Supplementary material for: CNNeoPP: a large language model-enhanced deep learning pipeline for personalized neoantigen prediction and liquid biopsy applications
Source: Front Immunol. 2026 Feb 4;17:1722117. doi: 10.3389/fimmu.2026.1722117 (PMC12913462; doi:10.3389/fimmu.2026.1722117)
Supplement: Supplementary file 2 [file DataSheet2.pdf]

Supplementary Figures

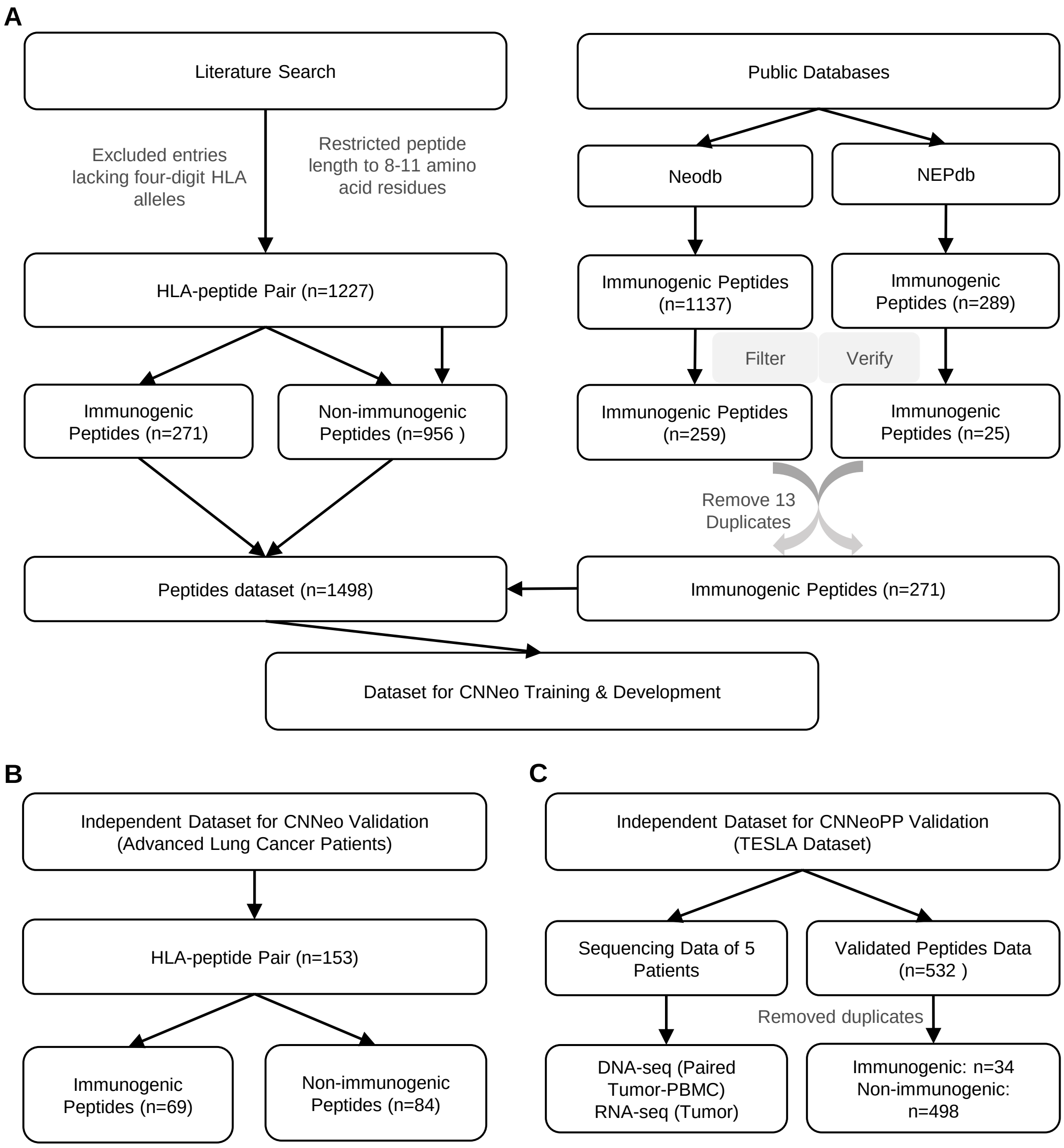

Supplementary Figure S1

Workflow of public dataset collection and consolidation in this study. **(A)** Datasets used for CNNeo training and development (Training dataset). **(B)** Independent dataset for CNNeo validation (Advanced lung cancer patients). **(C)** Independent dataset for CNNeoPP validation (TESLA dataset).

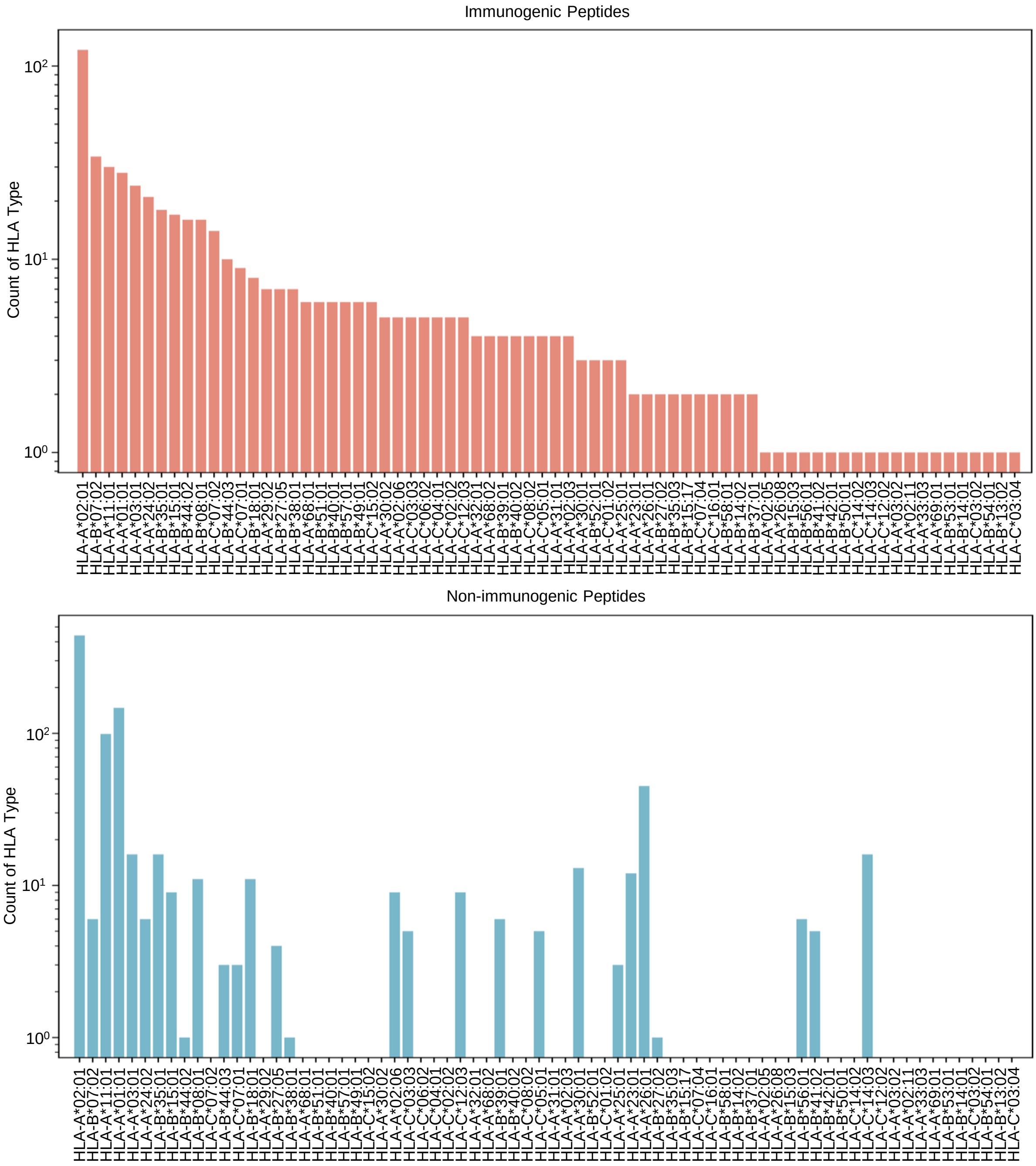

Supplementary Figure S2

Distribution of HLA genotypes paired with immunogenic (upper) and non-immunogenic (lower) peptides from Training dataset.

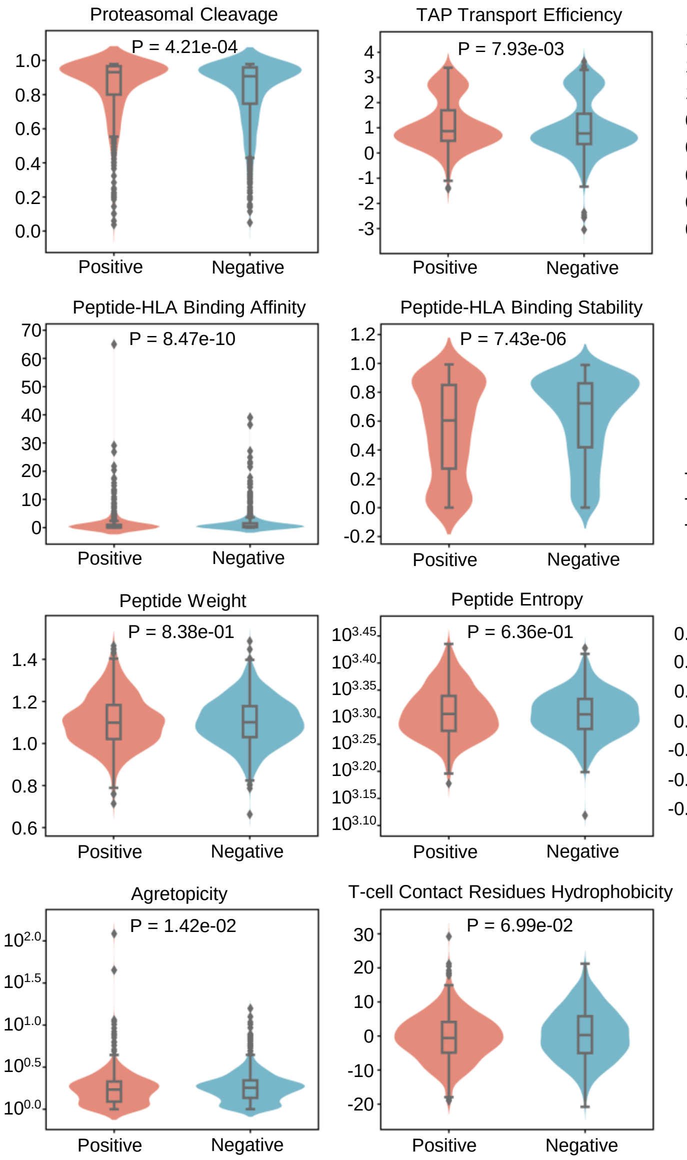

Supplementary Figure S3

Comparative visualization of 11 immunogenicity features between immunogenic (Positive) and non-immunogenic (Negative) datasets. Violin plots represent kernel density estimation, where wider sections indicate higher probability density. Box plots within the violins display the second and third quartiles with the interquartile range (IQR), with the median shown as a line inside the box. Whiskers extend to the first and fourth quartiles $\pm$ 1.5 $\times$ IQR or the minimum/maximum values within this range. Statistical significance was assessed using the Mann-Whitney U test, with p-values indicated in the plots.

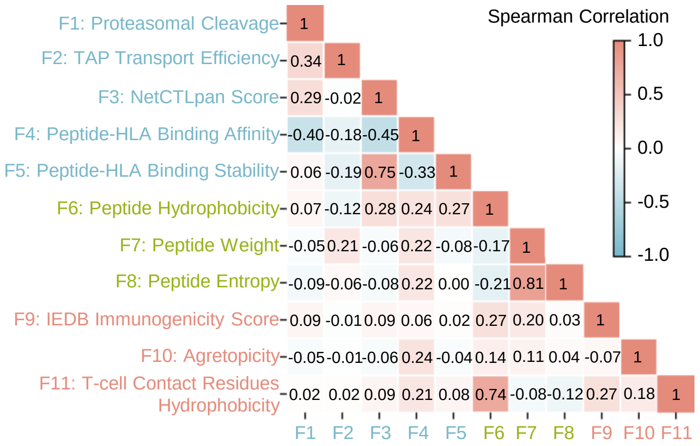

Supplementary Figure S4

Spearman correlation coefficient matrix of 11 immunogenicity features used in CNNeo. The color intensity reflects the absolute value of the Spearman Correlation Coefficient, indicating the strength of positive or negative correlations. Feature names are color-coded to indicate their respective categories: Neoantigen processing and presentation (F1-F5), Biochemical properties of neoantigens (F6-F8), and T cell recognition (F9-F11).

A

	PBMC 1	PBMC 2
Patient ID	BCa	LC1 & LC2
Tested Peptides	#1 - #21	#22 - #50
Viable Cell Count per mL	2.74×10^7	6.91×10^6
Total Cell Count per mL	2.84×10^7	7.28×10^6
Viability Percentage	96.39%	94.85%

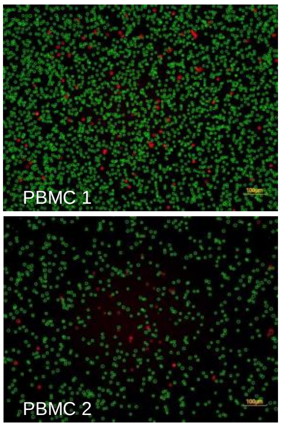

B

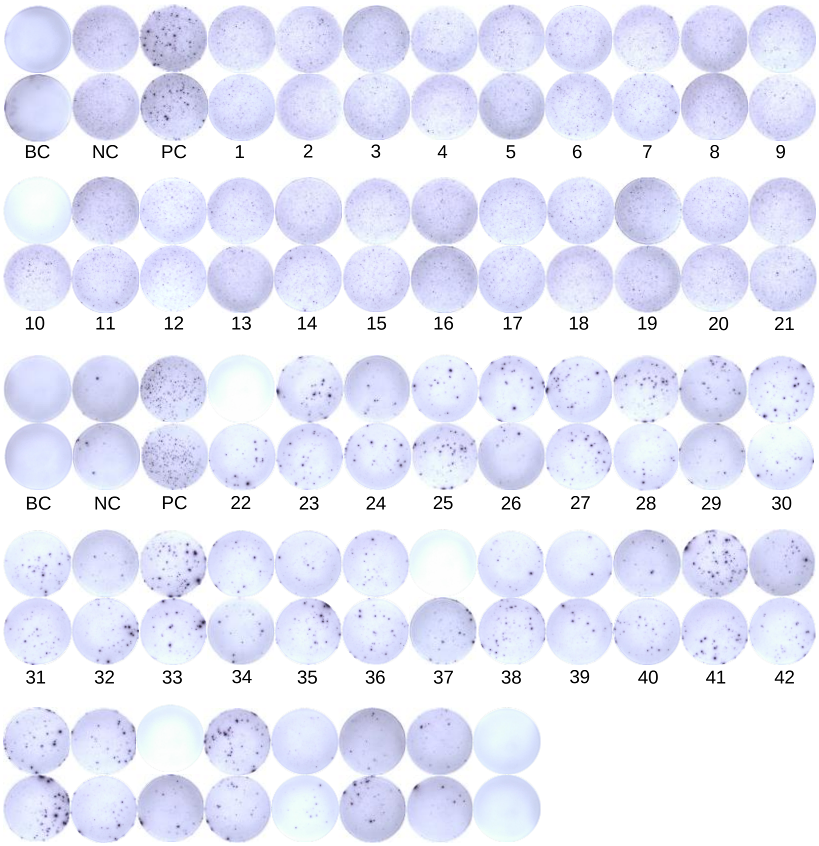

Supplementary Figure S5

Quality control of PBMCs and ELISpot validation of neoantigen responses. **(A)** Quality control assessment of PBMCs used in the experimental validation of CNNeoPP. A QC table is included, summarizing the total cell count, viable cell count, and cell viability percentage. Live/dead staining of PBMCs is shown, where green represents live cells and red represents dead cells. **(B)** ELISpot validation of neoantigen responses in three cancer patients. Additional replicate images are provided to complement the representative results shown in Figure 5B. Blank control (BC), positive control (PC) and negative control (NC) groups were included.

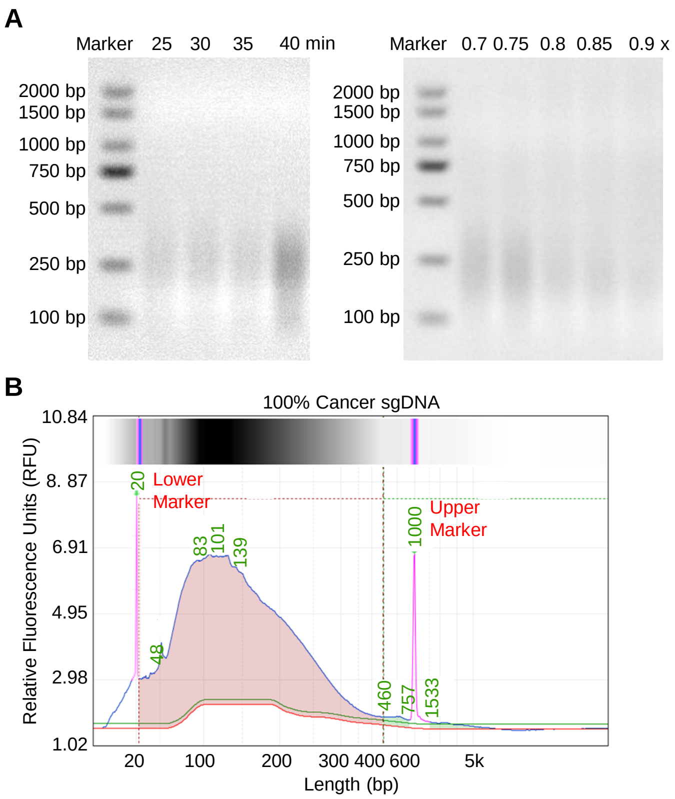

Supplementary Figure S6

Optimization tests for the preparation of cell-line derived cancer sgDNA samples. **(A)** Agarose gel electrophoresis comparing DNA fragment sizes across various enzymatic digestion time points (left) and during size selection using different AMPure XP bead-to-sample volume ratios as annotated (right). The optimized conditions were determined to be 40 minutes of digestion and a 0.80× volume ratio. **(B)** Fragmentation profiles of sheared genomic DNA (sgDNA) derived from cancer cell line.

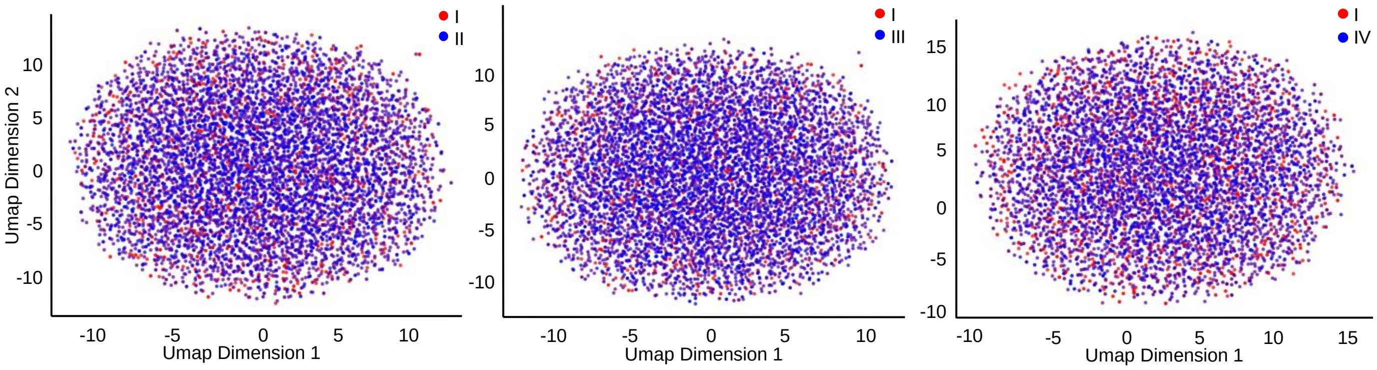

Supplementary Figure S7

UMAP visualization and comparison between 100% cancer sgDNA at 200× coverage with empirical 15% cancer cfDNA at 1000× and 200×, as well as in silico 15% cancer cfDNA at 200×, illustrating overlay patterns based on sequence features. Points are color-coded according to experimental conditions to highlight data distribution.
